# Day and night: diurnal phase influences the response to chronic mild stress

**DOI:** 10.3389/fnbeh.2014.00082

**Published:** 2014-03-14

**Authors:** Shilan Aslani, Mazen R. Harb, Patricio S. Costa, Osborne F. X. Almeida, Nuno Sousa, Joana A. Palha

**Affiliations:** ^1^Life and Health Sciences Research Institute (ICVS), School of Health Sciences, University of MinhoBraga, Portugal; ^2^ICVS/3B's - PT Government Associate LaboratoryBraga/Guimarães, Portugal; ^3^NeuroAdaptations Group, Max Planck Institute of PsychiatryMunich, Germany

**Keywords:** behavioral test, chronic mild stress, depression model, diurnal phase, stress

## Abstract

Chronic mild stress (CMS) protocols are widely used to create animal models of depression. Despite this, the inconsistencies in the reported effects may be indicative of crucial differences in methodology. Here, we considered the time of the diurnal cycle in which stressors are applied as a possible relevant temporal variable underlying the association between stress and behavior. Most laboratories test behavior during the light phase of the diurnal cycle, which corresponds to the animal's resting period. Here, rats stressed either in their resting (light phase) or active (dark phase) periods were behaviorally characterized in the light phase. When exposure to CMS occurred during the light phase of the day cycle, rats displayed signs of depressive and anxiety-related behaviors. This phenotype was not observed when CMS was applied during the dark (active) period. Interestingly, although no differences in spatial and reference memory were detected (Morris water maze) in animals in either stress period, those stressed in the light phase showed marked impairments in the probe test. These animals also showed significant dendritic atrophy in the hippocampal dentate granule neurons, with a decrease in the number of spines. Taken together, the observations reported demonstrate that the time in which stress is applied has differential effects on behavioral and neurostructural phenotypes.

## Introduction

Stressful life events predispose individuals to a number of neuropsychiatric disorders, especially depression. Depression is a devastating disease with a high rate of relapse for which there are still no effective treatments for more than 30% of the patients, despite decades of research (Kendler et al., 2001; Elizalde et al., 2010; Bartlang et al., 2012). The effects of stress depend on an interaction of multiple factors such as the quality (e.g., physical vs. psychological), intensity and chronicity of the applied stressor. Accordingly, the outcome of any particular stress paradigm can be potentially altered by very subtle procedural differences (Patchev and Patchev, 2006).

Chronic mild stress (CMS) is widely used to induce symptoms of depression in animals (Willner et al., 1992; Willner, 2005). Nonetheless, differences in the CMS protocol used by different researchers, resulting in a possible inadequacy or inappropriate use of the model, can likely be a contributor to the relatively poor rate of success in developing effective antidepressants. In agreement, the literature contains conflicting reports on the effects of CMS in terms of anhedonia, a key sign of depression (D'Aquila et al., 1994; Konkle et al., 2003; Grønli et al., 2005; Bessa et al., 2009a,b), and anxiety-like behaviors (D'Aquila et al., 1994; Gouirand and Matuszewich, 2005). These discrepancies are likely to result from methodological differences between different laboratories. An important variable that is usually not carefully described concerns the diurnal phase when CMS is applied. This is important because laboratory rodents are nocturnal and the salience of environmental stimuli, as well as the perception and response to such, may be a function of their periods of activity. In fact, when studying the effects of chronic restraint stress, Rybkin et al. (1997) and Perez-Cruz et al. (2009) concluded that the diurnal phase has an important impact on the observed behavioral phenotype. To the best of our knowledge, no study has been designed to specifically assess the effect of the diurnal phase in which stress is applied in the CMS model, which we address here.

## Materials and methods

### Animals

A total of 48 male Wistar rats (Charles-River Laboratories, Barcelona, Spain), 3 months old, were used in accordance with European Union regulations (Directive 86/609/EEC) and the National Institutes of Health guidelines on animal care and experimentation. Rats were housed (2 per cage) under standard laboratory conditions (room temperature 22°C; humidity 55%; food and water *ad libitum*). Animals were exposed to normal light cycle (lights on for 12 h starting at 08:00) or inverted light cycle (lights on for 12 h starting at 20:00). The normal light cycle was automatically controlled. The inverted cycle condition was attained by covering the animals' cage with black polypropylene (lightproof boxes; 48 × 30 × 46 cm) during the facility's light phase. The boxes were designed to assure proper ventilation and temperature maintenance similar to the standard laboratory cages. Animals in the inverted cycle condition were kept outside the black boxes in a separate room with lights on during the facilities' dark phase, and kept in inverted light condition 2 weeks before the start of the experimental procedures. Each group (normal light cycle vs. inverted light cycle) was further subdivided into 2 subgroups: control (not being disturbed besides handling) and CMS (exposed to a chronic mild stress protocol).

Animals were weighed once a week. Control rats were handled frequently during the experimental period and all groups were also handled for 5 min per day on the week before the start of the behavioral testing. Behavioral testing was performed 1 h after the start of the light phase of the diurnal cycle (animals resting period).

**Figure 1:**
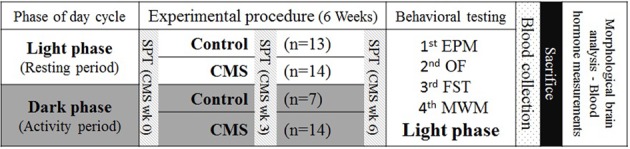
**Experimental timeline**. Rats were housed in different light phases and the CMS protocol was applied to both CMS groups at the same time. Behavioral tests were performed, in the order shown (third column), immediately after the CMS protocol (6 weeks), during the daily period of the light-dark cycle. SPT, sucrose preference test (at 3 time points); EPM, elevated plus maze; OF, open field; FST, forced swimming test; MWM, Morris water maze. Morphological and blood hormone analysis were performed after the sacrifice.

To avoid potentially confounding effects of different tests, these followed a specific order according to the sensitivity of each of them. As such, the following order was applied: elevated plus maze (EPM), open field (OF), forced swimming test (FST) and Morris water maze (MWM). Figure 1 depicts the scheme of the experimental approach and the order of the behavioral tests.

### Chronic mild stress (CMS)

The CMS protocol used was a slightly modified version of an unpredictable CMS protocol (Willner, 2005). Over a period of 6 weeks, it included a battery of chronic unpredictable mild stressors, namely: confinement to a restricted space for 2 h, placement in a tilted cage (30°) for 4 h, housing on damp bedding for 8 h, 12 h food deprivation followed by exposure to inaccessible food for 1 h, water deprivation for 12 h followed by exposure to an empty bottle for 1 h. The CMS was induced either in the light phase of the diurnal cycle (CMS-Light) or in the dark phase of the diurnal cycle (CMS-Dark).

### Sucrose preference test (SPT)

The SPT was performed in 3 time points throughout the CMS protocol: baseline (before the start of the CMS), in the middle (at the end of third week of the CMS) and at the end (at the end of last week of the CMS). After a 22 h food and water deprivation, animals were presented with two pre-weighed bottles containing 2% sucrose solution or tap water for 1 h. Sucrose preference was calculated according to the formula: sucrose preference = [sucrose intake/(sucrose intake + water intake)]^*^100. Anhedonia was defined as a reduction in sucrose solution consumption relative to baseline levels (Bekris et al., 2005).

### Forced swimming test (FST)

Learned helplessness, another dimension of depressive-like behavior, was evaluated with FST. Briefly, 24 h after a pre-test session (8 min), rats were placed in cylinders filled with water (25°C) to a depth such that the animals had no solid support. The actual test lasted for 5 min and was assessed using a camera. Learned helplessness behavior was defined as an increase in the time of immobility (defined as time spent either immobile or in movements to stay afloat) (Porsolt et al., 1978). An investigator blind to the experimental details scored the video recordings.

### Elevated plus maze (EPM)

The EPM was used to test anxiety-like behaviors. The EPM apparatus was made of a black polypropylene plus shaped platform (ENV- 560; Med Associates Inc, St Albans, VT, USA), which was elevated 72.4 cm above the floor and consisted of two opposite open (50.8 × 10.2 cm) and closed arms (50.8 × 10.2 × 40.6 cm). Rats were placed individually in the center of the maze and their ambulation was monitored online with an infrared photobeam system over a period of 5 min (MedPCIV, Med Associates Inc.). The ratio of the time spent in the open versus closed arms was used as an index of anxiety-like behavior.

### Open field (OF)

Locomotor and exploratory behaviors were investigated using the OF test. Briefly, rats were placed in the center of a brightly illuminated arena (Med Associates Inc.) and were allowed to explore it for 5 min. Exploration and the time and distances in the central and pre-defined peripheral areas were recorded online by two 16-beam infrared arrays. Total distances traveled were calculated as indicators of locomotor activity.

### Morris water maze (MWM)

An evaluation of cognitive function was performed in spatial working and reference memory tasks and in a reverse learning task in the MWM, as described previously (Cerqueira et al., 2007a). The MWM test was conducted in a circular black tank (170 cm diameter; depth: 50 cm) filled with water (23°C, around 30 cm depth) and placed in a dimly lit room with extrinsic visual clues. The tank was divided into imaginary quadrants and a hidden platform (12 cm diameter, submerged 2 cm below the surface of the water) was placed in the center of one of them. Data were collected using a video-tracking system (Viewpoint, Champagne au Mont d'Or, France). The spatial working memory test (Morris, 1984) was assessed in 4 consecutive days (4 trials per day, maximum of 2 min per trial). Test sessions begun with rats being placed in the tank, facing the wall of the maze, at a different starting point (in one of the imaginary quadrants in each session) and finished once the platform was found or if 2 min had elapsed (thereafter the animal was gently guided to the platform). On each trial day, the position of the platform was kept constant, but it was varied on each successive day such that all four quadrants were used. The distance traveled and the time spent to reach the platform (escape latency) was evaluated.

After assessing working memory, animals were tested for spatial reference memory. For this, the platform remained in the same quadrant to ensure that the animals correctly learned the position of the platform before assessment of reversal learning (further confirmed in the probe test). All of the remaining procedures were similar to the ones described for the working memory task. For the reverse learning task, after the animals learned the position of the platform, the escape platform was positioned in a new (opposite) quadrant and rats were tested in a 4-trial paradigm, as described above. For this task, distance and time spent swimming in each quadrant were recorded. The difference between distances traveled in the quadrant containing the newly positioned platform (“new”) and the quadrant that previously contained the platform (“old”) was calculated as a measure of reversal performance. In the water maze paradigm, the daily trial-to-trial progression of the distance swum to reach the platform was averaged for the different platform locations, whereas in the reference memory, day-to-day progression was averaged across the 4 daily trials for the same platform location.

### Corticosterone determinations

Blood samples, from each rat, were collected every 6 h during 24 h, by lancing the tip of the rats' tail and collecting blood drops into a microtube. Serum was obtained and stored at −80°C for later analysis. Serum corticosterone levels were determined by radioimmunoassay (MP Biochemicals, Costa Mesa, CA).

### Morphological analysis

Rats were perfused transcardially with 0.9% saline under deep anesthesia and the brains processed according to the protocol described by Gibb and Kolb (1998) for the Golgi analysis. Briefly, brains were immersed in Golgi-Cox solution (1:1 solution of 5% potassium dichromate and 5% mercuric chloride diluted 4:10 with 5% potassium chromate) (Glaser and Van der Loos, 1981) immediately after perfusion and remained for 20 d. Brains were subsequently transferred to a 30% sucrose solution (3 d), before being cut by vibratome. Coronal sections (200 μm thick) were collected in 6% sucrose and blotted dry onto cleaned, gelatin-coated microscope slides. They were subsequently alkalinized in 18.7% ammonia, developed in Dektol (Kodak, Rochester, NY, USA), fixed in Kodak Rapid Fix, dehydrated through a graded series of ethanol, cleared in xylene, mounted and cover slipped.

For the dendritic analysis of hippocampal dentate granule cells, neurons were chosen based on the following criteria: (1) cells being isolated from surrounding neurons, (2) full impregnation of the neurons, (3) cells location in the dentate gyrus region of the hippocampus (DG), and (4) no morphological changes attributable to incomplete dendritic impregnation of Golgi-Cox staining. For each selected neuron, all branches of the dendritic tree were reconstructed and the spine shape and numbers were determined at ×100 magnification using a motorized microscope (Axioplan 2, Carl Zeiss, Germany), attached to a camera (DXC-390, Sony Corporation, Tokyo, Japan), and the Neurolucida software (MicroBrightField Bioscience, Magdeburg, Germany). In order to minimize selection bias, slices containing the region of interest were randomly searched and the first 10 neurons fulfilling the above criteria were selected from each brain (Cerqueira et al., 2007b). The number of different dendritic spines was estimated by counting different spine shapes, namely thin, thick, ramified, and mushroom, in segments of the dendrites of dentate granule cells. After establishing the density of spines per category, their total number was calculated for each neuron [(number of spines/dendritic length)^*^total dendritic length].

### Statistical analysis

Homogeneity of the data was assessed before statistical analysis was performed. For the data on body weight changes, spatial reference and working memory in MWM, and Sholl analysis of dendrites in the DG, repeated measures ANOVA was used; different time points in corticosterone analysis were evaluated by *t*-test. One-Way ANOVA analysis was performed for EPM, OF, FST, SPT, reverse learning and probe task in the MWM, dendrite length and spine numbers of DG neurons; Bonferroni *post-hoc* analyses were used for comparing differences between the experimental groups. Results are expressed as means ± s.e.m. and statistical significance was accepted for *P* < 0.05. Behavioral and morphological analyses were done in two independent sets of animals. Since no statistical differences were found between these two groups (data not shown), the two sets of animals were grouped; therefore, figures and statistical analysis result from the combination of both sets of animals.

## Results

Comparison of the two control groups (normal and inverted light) did not reveal significant differences (Table 1). Therefore, with exception for the analysis of CORT determinations, the subsequent statistical analyses were performed by merging control data in a single group (CON), for clarity of comparison.

**Table 1:**
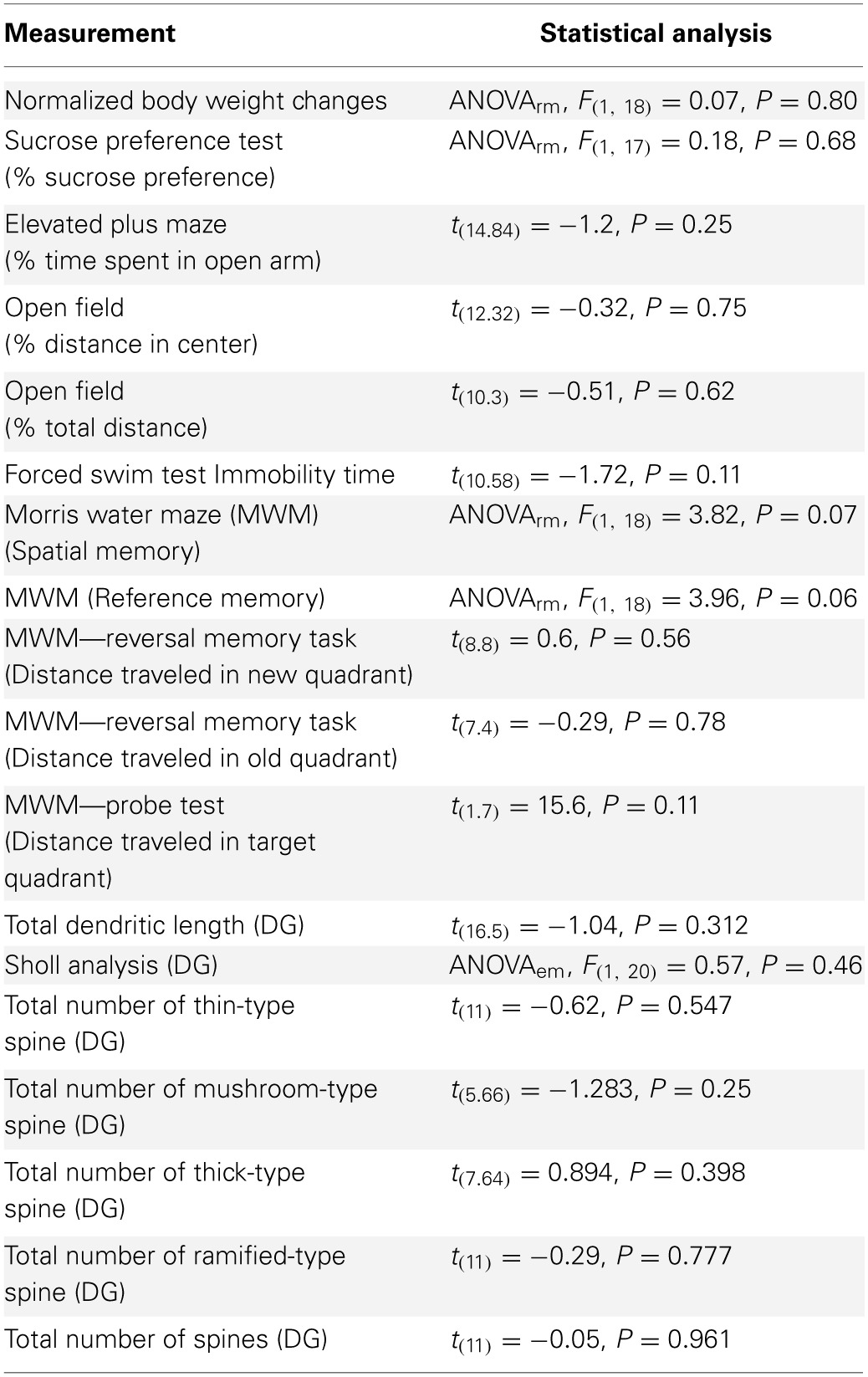
**Statistical analysis of two control (normal and inverted light) groups together**.

*Repeated measurements and t-test analysis indicate that there are no significant differences between the two control groups in any of the measurements*.

**Figure 2:**
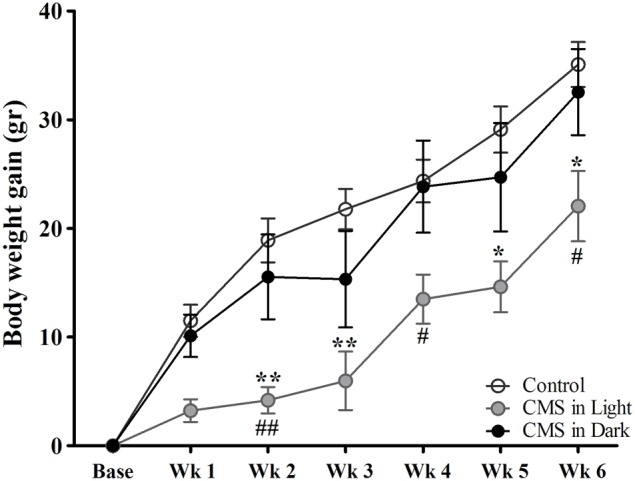
**Body weight gain during the CMS protocol**. Controls, *n* = 20, each CMS group, *n* = 14; ^*,#^*P* < 0.05. ^**,##^*P* < 0.01. Data presented as mean ± s.e.m. ^*^ indicates a significant difference between the CMS-Light and the CON group and ^#^ show a significant difference between the CMS-Light and CMS-Dark. There were no significant differences between the CMS-Dark and the CON group.

All groups of animals showed gains in body weight over the course of the experiment (Figure 2). A significant difference in body weight gain was observed between groups [*F*_(2, 45)_ = 4.99, *P* = 0.011]. Animals who were exposed to CMS in the light phase of the diurnal cycle (CMS-Light) gained remarkably less weight than the CON group during the stress exposure period (*P* = 0.015). Interestingly, this difference was not observed in animals exposed to stressors in their dark phase of the diurnal cycle (CMS-Dark) (*P* = 0.98); consequently, the CMS-Light and CMS-dark groups also showed significant differences (*P* = 0.042).

**Figure 3:**
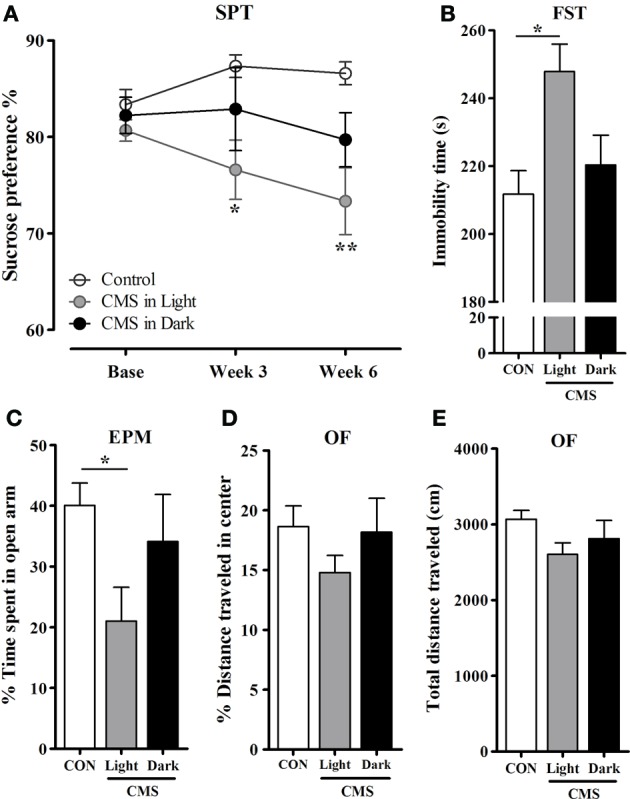
**Emotional behavior in rats exposed to CMS in different diurnal phases. (A)** Preference changes of sucrose consumption in the Sucrose preference test (SPT). ^*^ indicates a significant difference between the CMS-Light and CON. There were no significant differences between the CMS-Dark and the two other groups. **(B)** Forced swimming test (FST). Immobility time during the test period. **(C)** Elevated plus maze (EPM). Percentage of time spent in the open arms **(D–E)** Open field test (OF). **(D)** Percentage of the distance traveled in the center and **(E)** Total distance traveled in the OF apparatus. Controls, *n* = 20, each CMS group, *n* = 14; ^*^P < 0.05; ^**^P < 0.01. Data presented as mean ± s.e.m.

Anhedonia was evaluated by the sucrose preference changes relative to individual's baseline preference. The pattern indicated a reduction in sucrose solution consumption after exposure to CMS [*F*_(2, 44)_ = 4.50, *P* = 0.017]. However, this decrease was only significant in the CMS-Light animals when compared to CON (CMS-Light/CON: *P* = 0.014; CMS-Dark/CON: *P* = 0.358; CMS-Light/CMS-Dark: 0.606) (Figure 3A). In accordance, immobility time in the FST was remarkably increased in the CMS-Light group [*F*_(2, 45)_ = 4.49, *P* = 0.017; CMS-Light: *P* = 0.018 when compared to CON] indicating the development of depressive-like behavior. Such phenotype, however, was not observed in CMS-Dark animals (CMS-Dark/CON = 0.99; CMS-Dark/CMS-Light; 0.089) (Figure 3B).

Anxiety assessment in the EPM test indicated a CMS effect on the time spent in open arms in different groups [*F*_(2, 44)_ = 3.30, *P* = 0.046]. Nevertheless, only the CMS-Light group spent significantly less time in the open arms compared to CON animals (*P* = 0.043). The CMS-Dark rats did not differ in this parameter from CON (*p* = 0.99) or CMS-Light (*p* = 0.36) (Figure 3C). In the OF test, no differences were observed between group in distance traveled in the center of the arena [*F*_(2, 40)_ = 1.07, *P* = 0.353] (Figure 3D) and in total distances traveled [*F*_(2.39)_ = 2.77, *P* = 0.075] (Figure 3E). The former parameter is a measure of anxiety-like behavior, whereas the latter is an index of locomotor behavior.

**Figure 4:**
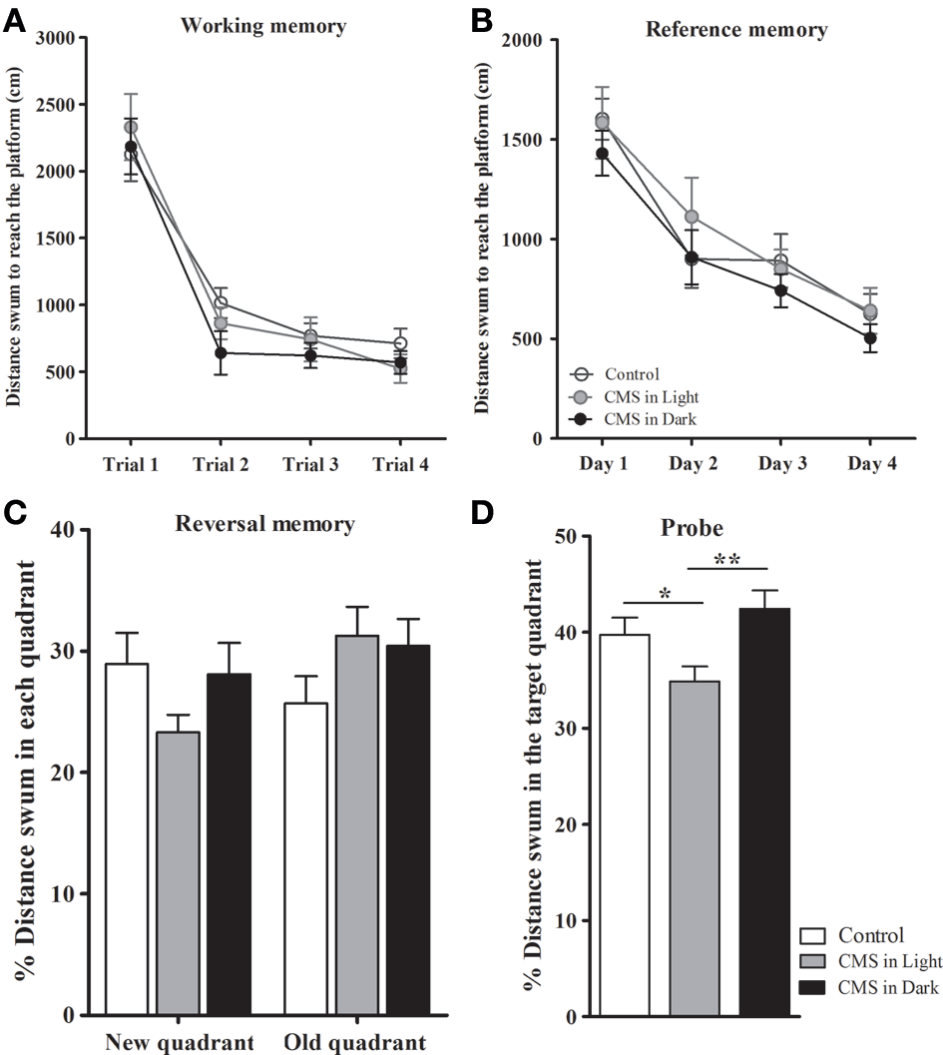
**Impact of CMS during different phases of the day light-dark cycle on spatial working, reference and reversal memory. (A)** Average distance swum during the spatial working memory task. **(B)** Average distance swum during the reference memory task. **(C)** Percentage of distance swum in the new/old quadrant during the reversal memory task. **(D)** Percentage of distance swum in the target quadrant during the probe test. Controls, *n* = 20, each CMS group, *n* = 14; ^*^P < 0.05. ^**^P < 0.01. Data presented as mean ± s.e.m.

The learning curve in spatial working (Figure 4A) and reference (Figure 4B) memory tasks in the MWM did not show differences between groups [*F*_(2, 45)_ = 1.26, *P* = 0.294; *F*_(2, 43)_ = 1.11, *P* = 0.338 respectively]. Similarly, no differences were observed in the reversal learning task performance (a test for behavioral flexibility measurement) [distance swum in the “new” quadrant: *F*_(2, 44)_ = 2.04, *P* = 0.142; distance swum in the “old” quadrant: *F*_(2, 43)_ = 1.82, *P* = 0.175] (Figure 4C). The significant difference in the analysis of the probe test was due to considerably decrease in the distance swum in the target quadrant in CMS-Light animals [*F*_(2, 41)_ = 8.23, *P* = 0.001; *P* = 0.043] when compared to the CON group; this difference was also observed between CMS-Light and the CMS-Dark groups (*P* = 0.001). The CMS-Dark group however, did not differ from the CON group (*P* = 0.246) (Figure 4D).

**Figure 5:**
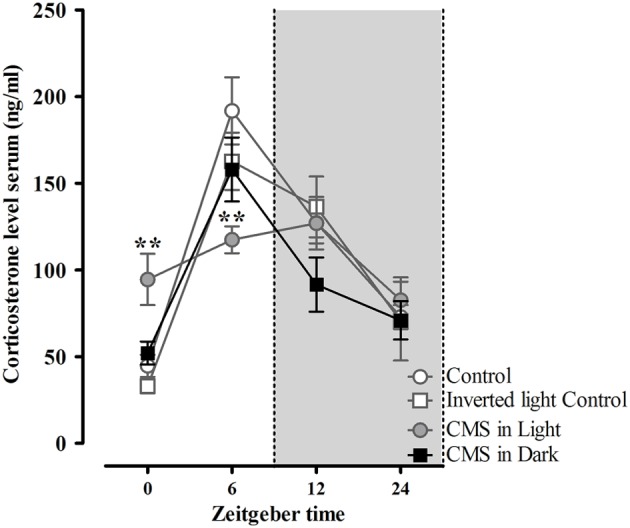
**Diurnal rhythm of corticosterone secretion in rats exposed to CMS in different phases of the day light-dark cycle**. Serum corticosterone levels during 24 h post CMS, measured every 6 h. Controls, *n* = 20, each CMS group, *n* = 14; ^**^P < 0.01. Data presented as mean ± s.e.m. ^**^ indicate significant differences between the CMS-Light and the CON group.

**Figure 6:**
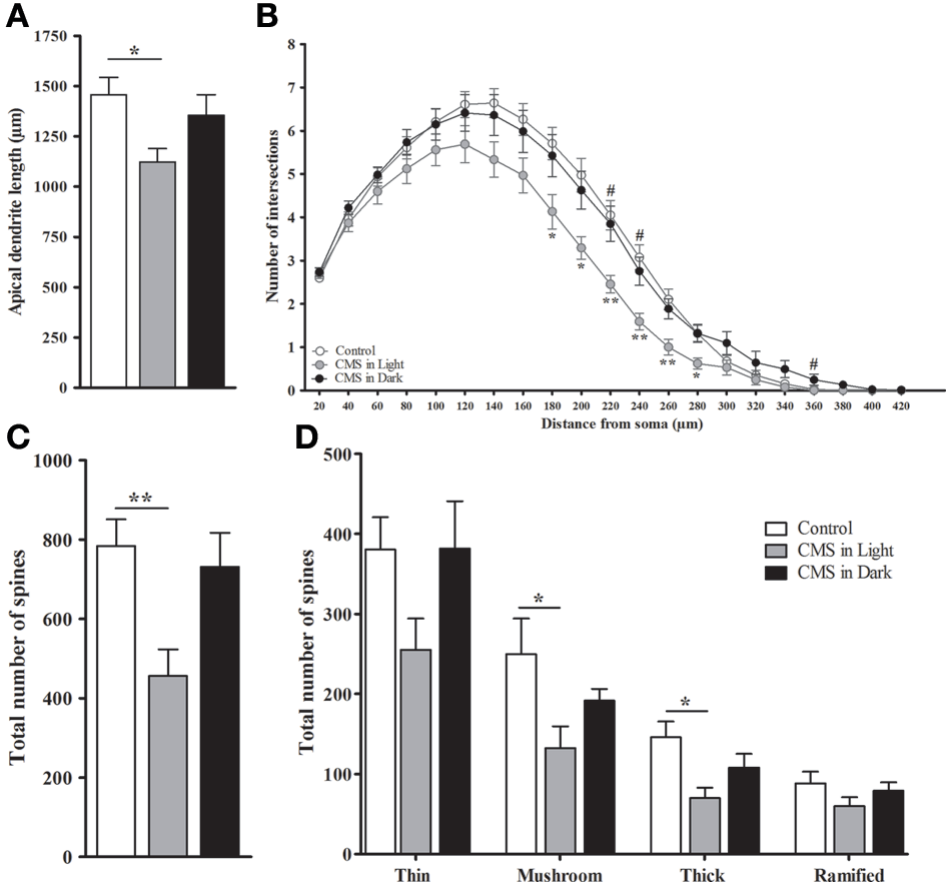
**Morphometric analysis of dendritic arborizations in the hippocampal dentate gyrus. (A)** Total dendritic length. **(B)** Sholl analysis-derived distribution of dendrites. **(C)** Total number of spines. **(D)** Total number of different types of spines. Controls, *n* = 20, each CMS group, *n* = 14; ^*^P < 0.05. ^**^P < 0.01. ^#^*P* < 0.05. Data presented as mean ± s.e.m. ^*^ and ^**^ indicate significant differences between the CMS-Light and the CON group. ^#^ indicates a significant difference between the CMS-Light and the CMS-Dark group.

Indicating for different stress effects regarding different phases of the diurnal cycle, only the CMS-Light group revealed disrupted circadian rhythm on the corticosterone profile (when all groups were compared together). These group differences were found mostly in the animals' light phase, at zeitgeber time (Zt) 0 [*t*_(24)_ = −2.89, *P* = 0.008] and Zt 6 [*t*_(25)_ = 3.22, *P* = 0.004] which correspond to the animal's resting period (Figure 5). Morphological analysis in the DG revealed a decreased dendrite length in granule neurons of animals exposed to CMS [*F*_(2, 45)_ = 3.8, *P* = 0.03]; this difference again was only significant in the CMS-Light group (CMS-Light/CON: *P* = 0.026; CMS-Dark/CON: 0.99; CMS-Light/CMS-Dark: *P* = 0.3) (Figure 6A). This observation was confirmed by the Sholl and spine density analysis of the same neurons; with the Sholl data [*F*_(2, 45)_ = 4.36, *P* = 0.02] indicating a decrease in the number of intersections with significant group difference between the CMS-Light and other groups (CMS-Light/CON: *P* = 0.025; CMS-Dark/CON: 0.99; CMS-Light/CMS-Dark: *P* = 0.58) (Figure 6B).

Exposure to CMS led to a reduction in the overall spine number [*F*_(2, 28)_ = 5.59, *P* = 0.009] in the hippocampal dentate gyrus (Figure 6C). This decrease was observed only in the animals that were stressed in their resting period (CMS-Light/CON: *P* = 0.01; CMS-Dark/CON: 0.99; CMS-Light/CMS-Dark: *P* = 0.57). More specifically, a statistically significant decrease was found in two spine types, namely: mushroom spines [*F*_(2, 26)_ = 5.47, *P* = 0.01; CMS-Light/CON: *P* = 0.01; CMS-Dark/CON: 0.99; CMS-Light/CMS-Dark: *P* = 0.69], and thick spines [*F*_(2, 28)_ = 4.55, *P* = 0.02; CMS-Light/CON: *P* = 0.017; CMS-Dark/CON: 0.44; CMS-Light/CMS-Dark: *P* = 0.53]. This difference was not observed in thin [*F*_(2, 28)_ = 2.85, *P* = 0.075] and ramified spines [*F*_(2, 28)_ = 1.2, *P* = 0.317] (Figure 6D).

## Discussion

This study shows that the effect of exposure to CMS depends on the phase of the diurnal cycle in which the CMS procedure is applied. Depressive and anxiety-related behaviors and memory impairments only occur when rats are exposed to CMS during their daily period of rest (light phase). Similarly, CMS during the inactive, but not active, day period leads to body weight loss, altered corticosterone secretory profiles, and reduced dendritic arborization and number of mature (thick, ramified, and mushroom) spines in the granule cells of the hippocampus. These findings clearly show that rats can cope with CMS during their active period. Results are relevant when considering that most studies reported in the literature use animal models in which stress is induced during the resting phase. In addition, these observations should be taken into consideration when studies on animal models of CMS serve as basis to infer on the consequences of human exposure to stress.

Delayed body weight gain (in younger subjects) and weight loss are well known consequences of chronic stress (Barr and Phillips, 1998; Bielajew et al., 2002; Konkle et al., 2003). In the present study, CMS during the daily resting (light) period caused significant loss of body mass; the same treatment during the dark phase of the diurnal cycle resulted in only minor fluctuations in body mass. Results are in accordance with those reported by Grønli et al. (2005), which is one of the few laboratories, to our best knowledge, that has performed CMS in the dark phase of the light cycle. Previous studies have shown that disruption during the resting phase interferes with the pattern of food ingestion and metabolism (Nagano et al., 2003; Salgado-delgado et al., 2008, 2011), suggesting disruption of the circadian regulation of feeding and metabolism. Interestingly, glucocorticoid secretion normally follows a tight circadian rhythm, with peak secretion occurring just at the onset of the daily period of darkness when rodents show high levels of locomotor activity and feeding. Here we found the rhythmic secretion of corticosterone to be markedly altered in rats that had experienced CMS during their inactivity period (light phase). Specifically, the corticosterone profile of these animals showed a sluggish rise as the nocturnal period approached, and the zenith of secretion was shifted to the middle of the dark period of the day cycle. At the same time, these animals showed higher blood levels of corticosterone at the beginning of the daily period of light. In other words, CMS applied during the animals' inactivity period resulted in a blunted and phase-shifted corticosterone rhythm. These results largely concur with those previously reported (Bielajew et al., 2002; Konkle et al., 2003; Ushijima et al., 2006). Interestingly, similar disruption of the daily glucocorticoid rhythm is also seen in a large sub-group of patients suffering from major depression (Jarcho et al., 2013). It has been shown that the inability to mount an appropriate response to stress makes individuals more vulnerable to stressors (Zimmermann and Critchlow, 1967); therefore, it is likely that this disrupted profile is critical for explaining the deleterious effects of stress during the resting phase of the animals' diurnal cycle. Indeed, there is evidence that the lack of synchrony of the internal clock, in association to altered glucocorticoid levels, might play a role in the development of emotional disturbances, namely depression (Salgado-delgado et al., 2011), a finding that we observed exclusively in animals exposed to CMS during the light phase of the day cycle. However, we should consider that the present study has only assessed behavior during the resting phase of rodents. Future studies should evaluate the impact of the time of testing in the behavioral phenotype of stress rodents.

Learned helplessness and anhedonia, two characteristics of depressive illness, can be induced by CMS in animals (D'Aquila et al., 1994; Elizalde et al., 2008; Bessa et al., 2009a,b). These behaviors can be assessed in the SPT and FST, respectively; however, these have not always been consistently reproduced (Konkle et al., 2003). The time of the day may contribute to such discrepancies, particularly as the present study showed that only animals exposed to CMS in their resting period displayed signs of depressive-like behavior. A previous study (based on chronic restraint stress) also noted the importance of the diurnal phase in which the stress is applied in determining responses in the SPT and FST (Huynh et al., 2011), which is in accordance with the present findings. We used the OF test to address measures of anxiety. Grønli et al. (2005), who performed CMS exposure in dark phases, failed to find signs of increased anxiety, similar to our current observations; yet, on this parameter the study of restraint stress in rats observed the opposite effect (Huynh et al., 2011).

The effects of CMS on learning and memory in rodents are controversial. For example, while CMS-induced impairments in learning have been reported (Song et al., 2006; Elizalde et al., 2008), we failed to observe deficits in spatial reference learning and memory after this treatment (Bessa et al., 2009b; present study). On the other hand, as reported herein, significant cognitive impairments were detectable by the probe test in rats exposed to CMS solely during the daily period of light.

Finding anatomical correlates of behavioral changes may help to understand the mechanisms underlying the response to stress. Bessa et al. (2009a) showed that stress induces dendritic atrophy (length, branch number and spine numbers) in hippocampal granule neurons, an area implicated in spatial learning and memory, as we also show here in animals exposed to CMS during the daily period of inactivity (light). This reduction in the dendritic arbor and in the number of spines, particularly mature spines, is likely to translate into synaptic signaling decrease (Kennedy et al., 2005), and in impairments in hippocampal-based learning and cognition abilities (Bliss and Collingridge, 1993). Moreover, since the hippocampus exerts inhibitory control over the HPA axis, we suggest that these structural changes might also translate into reduced inhibitory input to the hypothalamus, thus contributing to the marked CMS-induced disruption of the corticosterone secretory profile.

The experiments reported here help clarify discrepancies in the literature regarding the robustness of the CMS paradigm for the induction of depressive- and anxiety-like behaviors in rats. Briefly, our results show that CMS is only effective when applied during the daily period of the diurnal cycle when animals are inactive, primarily sleeping. CMS administered during the daily period of activity does not trigger depressive- or anxiety-like behaviors and may lead to the false presumption that the method is invalid or that animals are resilient to the deleterious effects of stress.

### Conflict of interest statement

The authors declare that the research was conducted in the absence of any commercial or financial relationships that could be construed as a potential conflict of interest.
